# p300/CBP as a Key Nutritional Sensor for Hepatic Energy Homeostasis and Liver Fibrosis

**DOI:** 10.1155/2018/8168791

**Published:** 2018-05-15

**Authors:** Weilei Yao, Tongxin Wang, Feiruo Huang

**Affiliations:** Department of Animal Nutrition and Feed Science, College of Animal Science and Technology, Huazhong Agricultural University, Wuhan 430070, China

## Abstract

The overwhelming frequency of metabolic diseases such as obesity and diabetes are closely related to liver diseases, which might share common pathogenic signaling processes. These metabolic disorders in the presence of inflammatory response seem to be triggered by and to reside in the liver, which is the central metabolic organ that plays primary roles in regulating lipid and glucose homeostasis upon alterations of metabolic conditions. Recently, abundant emerging researches suggested that p300 and CREB binding protein (CBP) are crucial regulators of energy homeostasis and liver fibrosis through both their acetyltransferase activities and transcriptional coactivators. Plenty of recent findings demonstrated the potential roles of p300/CBP in mammalian metabolic homeostasis in response to nutrients. This review is focused on the different targets and functions of p300/CBP in physiological and pathological processes, including lipogenesis, lipid export, gluconeogenesis, and liver fibrosis, also provided some nutrients as the regulator of p300/CBP for nutritional therapeutic approaches to treat liver diseases.

## 1. Introduction

Obesity, type 2 diabetes mellitus (T2DM), and nonalcoholic fatty liver disease (NAFLD) are becoming major health concern in the world, and nutrients associated with those disease are grouped in the metabolic syndrome [1–3]. These diseases seem to have two common characteristics: they involve the destruction of homeostasis, and they are generally associated with chronic inflammation [4]. The liver is well known to be the largest visceral organ in the body and maintains the balance of glucose and lipids by adapting its metabolic activity to the needs of energy of the organism [5]. Recently multiple evidence indicates that nutrient excess and obesity activates several proinflammatory signaling pathways, leading to chronic low-grade inflammation, also called metaflammation in liver, which may give rise to the chronic liver injury. Sustained wound healing response to sustained chronic liver injury will lead to fibrosis. Advanced liver fibrosis leads to cirrhosis, liver failure [6, 7]. Further, the damage of hepatic delicate energy balance may aggravate many pathological states such as obesity, diabetes, and other chronic noncommunicable diseases.

With the development of epigenetics in liver disease, it has received considerable research interest in the past 20 years. The disorder of energy metabolism is considered to be mediated through epigenetic mechanisms, by altering the expression of key genes [8]. The most well studied epigenetic marks is posttranslational modifications (PTMs) of histones. During the past decades, specifically, the inventory of acetylation, as evidenced by the number of modification sites identified, is fast catching up with other major PTMs such as phosphorylation and ubiquitylation. Acetylation is a highly reversible process that is regulated by histone acetyltransferases (HATs) and histone deacetylases (HDACs), respectively [9, 10]. Recently, acetylation status of nonhistone proteins has also been shown to be regulated by HATs and HDACs [11, 12]. Predictably, as the pivotal metabolic organ, liver is largely subjected to lysine acetylation. The acetylation of the nonhistone proteins including transcription factors involved in regulation of metabolic genes and their relevance to metabolic homeostasis are even more important.

p300/CBP has a histone acetyltransferase activity that transfers an acetyl group to the lysine residue, and the acetylation level of nonhistone proteins has been identified as a key mechanism for regulating transcription [13–16]. Recently, the roster of p300/CBP acetylated lysine sites has expanded, through using multifarious nutritional, genetic, and pharmacological model systems. Emergent theory indicates that high glucose and lipid as well as metabolic hormone alteration may regulate the activity of p300/CBP, and it is also involved in regulating multiple hepatic energy homeostasis and inflammatory pathway in response to nutrient excess or deprivation, through acetylating nonhistone proteins including SREBP1C (K289 and K309), ChREBP (K672), and FOXO1 (K242, K245, and K262). Besides, p300/CBP is global transcriptional coactivators that are involved in the expression of lipogenesis and gluconeogenesis genes via regulating DNA binding transcriptional factors. Here we will focus primarily on the different mechanisms, that is, p300/CBP in regulating hepatic energy homeostasis and liver fibrosis under different trophic conditions.

## 2. p300/CBP in Hepatic Energy Homeostasis

p300 and CBP are two acetyltransferase enzymes in humans and most higher eukaryotes. p300 (also called EP300 or KAT3B) is so-named because it is about 300 kDa in size (with 2414 amino acids). CBP (also called CREBBP or KAT3A) is composed of 2441 amino acids, and because of the high sequence homology observed between it and p300, the two proteins are now collectively referred to as p300/CBP [28]. p300/CBP plays also a role in pivotal transcriptional coactivator proteins in integrating and coordinating multiple signal-dependent events [29]. p300 and CBP are crucial regulators of hepatic homeostasis through both their acetyltransferase activity and transcriptional coactivator. More recently, a growing number of experiments have suggested p300/CBP in hepatic metabolic homeostasis including lipogenesis, gluconeogenesis, and the regulation of insulin action. These results indicate that p300/CBP activity might insure the coordinated regulation of several different metabolic functions in liver. Further elucidation of the role p300/CBP plays in hepatic energy homeostasis may provide novel insights into developing treatments for hepatic metabolic syndrome and other liver diseases.

## 3. Hepatic Lipid Metabolism

### 3.1. Hepatic De Novo Lipogenesis

The balance between lipid synthesis and catabolism is closely associated with the nutritional status of the organism [30, 31]. Condition of overnutrition promotes lipid synthesis while malnutrition accelerates fatty acid oxidation [32]. Although lipids are primary for maintaining organismal homeostasis, many diseases, such as, obesity, nonalcoholic fatty liver disease, and type 2 diabetes are associated with the disordered lipid synthesis [33]. An amount of literature has implicated that p300/CBP affects the lipid metabolism in different tissues and cells; here we primarily clarify its effects on hepatocyte and liver.

Recently, by treating with glucose and insulin to mimic feeding conditions in HepG2 cells, Ponugoti et al. found that hepatic de novo lipogenesis was increased [20]. Remarkably, hepatic de novo lipogenesis levels were also highly elevated in diet induced obese mice [20]. Sterol regulatory element-binding transcription factor 1 (SREBP1C) is a critical lipogenic activator, which is dynamically regulated in response to nutritional changes [34–36]. It was taken for granted that SREBP1C expression levels in liver were increased in response to feeding. SREBPs are bHLH-LZ transcription factors that bind as dimers to sterol regulatory elements of target genes for lipid metabolism. By overexpression of SREBP1C in the mice livers, SREBP1C has been shown as a crucial role in the transcriptional activation of lipogenic genes, which led to increase of hepatic lipid accumulation [37, 38]. The activity of SREBP1C was shown to be regulated by posttranslational modifications, such as phosphorylation, ubiquitination, and acetylation [39]. The authors observed that SREBP1C was acetylated at Lys-289 and Lys-309 by p300/CBP through tandem mass spectrometry [40]. So, p300/CBP may regulate lipid synthesis via acetylation of SCREBP1C. Under high glucose and insulin conditions, SREBP1C was acetylated by p300/CBP acetyltransferase in both cells and mouse liver, which led to increasing its stability and recruitment to its lipogenic target gene promoters [20]. Those findings have crucial suggestions in the context of metabolic regulation, as inhibition of p300/CBP-dependent SREBP1C acetylation may provide a measure to reduce lipogenesis during excess nutrient. In addition, small molecules that inhibit the acetylation of SREBP1C by inhibiting p300/CBP may be used to treat metabolic disorders, such as NAFLD, obesity, and T2DM.

The liver increases fatty acid synthesis in response to glucose and insulin by upregulating the expression of lipogenic genes such as fatty acid synthase (FAS) and acetyl-CoA carboxylase (ACC) through the SREBP1C and carbohydrate-responsive element-binding protein (ChREBP) [41]. ChREBP was also shown as a major regulator of lipogenesis and acts in synergy with SREBP1C to activate fatty acid synthesis [42, 43]. The targets of ChREBP include ATP citrate lyase (ACLY), FAS, ACC, and stearoyl-CoA desaturase 1 (SCD1) [44, 45]. ChREBP is also regulated by posttranslational modification. During fasting, ChREBP could be phosphorylated at S196 and T666 by protein kinase A (PKA), leading to prevention of nuclear entry and reducing its DNA binding, respectively [46, 47]. Under high glucose uptake conditions, ChREBP was dephosphorylated to be converted into an active form, which is displaced into the nucleus to facilitate binding of ChREBP to its response element [47]. Julien and colleagues found that ChREBP was acetylated at Lys-672 by glucose-activated p300/CBP, which enhanced its transcriptional activity by increasing its recruitment of its target gene promoters in cultured mouse hepatocytes [19]. Furthermore, in mice models of obesity and T2DM, high p300/CBP HAT activity was associated with ChREBP hyperacetylation and hepatic steatosis [19]. In accordance with these results, the authors also recently showed that p300/CBP was a crucial regulator in regulating activity of nuclear bile acid receptor (FXR) through acetylating FXR in K217 acetylation site [17]. Remarkably, in high glucose and insulin states, the deacetylation of FXR was dramatically decreased, and hepatic FXR acetylation levels were highly increased [17, 18]. Acetylation of FXR enhanced its stability but inhibited heterodimerization with RXRa, DNA binding, and transactivation activity [17]. It was accepted that p300 acetylated FXR which decreased the FXR activity [17]. Inactivated FXR would aggravate hyperglycemia and hyperlipidemia via SHP expression. In addition, increased SHP RNA level leads to decreasing PLTP and VLDLR RNA levels and increasing G6Pase and PEPCK RNA levels [18]. So the acetylated FXR by p300 led to a decreased activity of FXR and increased expression of SHP which promoted target genes expression associated with lipid and glucose metabolism. Later, SIRT1 deacetylated FXR, which decreased FXR acetylation levels. In normal conditions, FXR is activated by agonists and p300 is recruited, which results in increased acetylation of histone H3 and, subsequently, transcriptional activation. At the same time, the process for limiting the stimulated activity is initiated by acetylation of FXR by p300, which impairs FXR interaction with RXRa and DNA binding, resulting in dissociation of FXR from the promoter. The activity of FXR is tightly balanced by the opposing actions of p300 and SIRT1 via FXR and histone acetylation. However, in metabolic disease states, deacetylation of the acetylated FXR may be inhibited because of low expression and activity of SIRT1 [17]. These findings suggested that inhibition of hepatic p300 activity may be beneficial for treating metabolic diseases.

**Figure 1:**
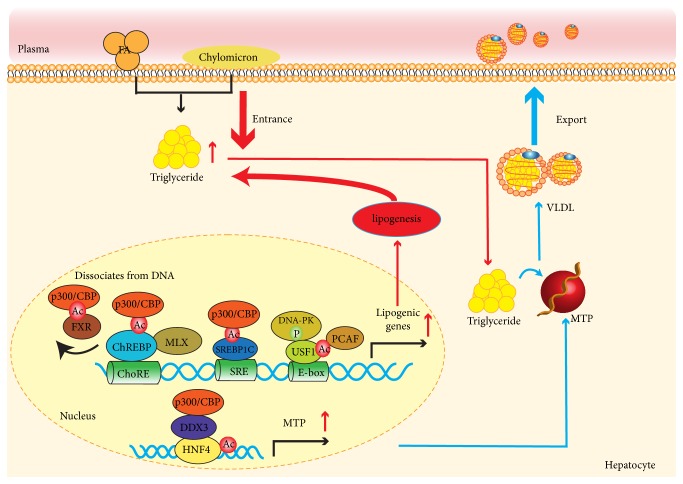
*Potential contributions of p300/CBP to development of hepatic lipid metabolism. Red arrow*: hepatic de novo lipogenesis and hyperlipidemia in p300/CBP regulation: the scheme indicates the regulatory actions of the inducible p300/CBP acetylation and their effects on de novo lipogenesis in both enzymatic and transcriptional events.* Blue arrow*: the regulation of p300/CBP in lipid export by interacting with DDX3 and inducing acetylation of HNF4 which increased the MTP promoter activation (HNF4: hepatocyte nuclear factor 4; MTP: microsomal triglyceride transfer protein).

Hepatic lipid synthesis is modulated in a precise manner in response to change of nutrients and hormones [48]. Transcription factors for lipogenic genes (such as FXR, SREBP1C, and ChREBP), are equally activated on insulin and glucose signaling which involved multiple downstream molecules, including a variety of kinases and phosphatases [48]. p300/CBP could acetylate these transcription factors under high glucose and insulin conditions, leading to the activation of lipogenic genes transcription. The work discussed here demonstrates that various transcription factors involved in acetylation of p300/CBP may provide a therapeutic targets for suppression of activity of p300/CBP to combat overnutrition (Figure 1).

### 3.2. Hepatic Lipid Export

Lipid homeostasis is basically maintained through the balance between inflow (lipogenesis) and outflow (*β*-oxidation and lipid export) of lipid metabolism, which is under complicated and sophisticated controls [21]. Blocking of lipoprotein assembly may inhibit lipid export, which is one of the causes of lipid accumulation, therefore resulting in the occurrence of hepatic steatosis [21]. Microsomal triglyceride transfer protein (MTP), a crucial regulator of liver lipid homeostasis, is synthesized largely in the liver and intestine, which is primarily modulated at the transcriptional level [49]. The crucial role of MTP in the assembly and secretion of lipoproteins was originally identified in patients with abetalipoproteinemia, a syndrome caused by mutations in MTP gene4 [50] and observed in MTP liver-specific knockout mice [51]. Recently, RNA helicase DDX3, also known as DBX or CAP-Rf [52–54], has been reported to be associated with the expression of MTP which in turn leads to lipid accumulation and affects ApoB secretion [21, 55, 56].

At present, there is limited information between p300/CBP and lipid export. Tsai and colleagues showed that downregulation of DDX3 led to lipid accumulation and reduced ApoB secretion in hepatocyte-derived HuH7 or HepG2 cell lines [21]. The mechanism of this effect appears to be related to the interaction of p300/CBP with DDX3. Further research demonstrated that DDX3 interacted with p300/CBP and induced acetylation of hepatocyte nuclear factor 4 (HNF4) which synergistically increased the MTP promoter binding affinity of HNF4 [57–60]. Deservedly, many approaches had focused on MTP as a therapeutic candidate in virtue of its physiological importance of lipoprotein secretion [21, 61]. However, this observation demonstrated a novel role of p300/CBP in lipid metabolism and provided a clue to design new strategies for therapy of hepatosteatosis (Figure 1).

## 4. Hepatic Glucose Homeostasis

Glucose serves as a primary fuel for mammals [23]. Maintaining the glucose balance is one of the central mechanisms for the survival of the organisms [62]. The liver is crucial in maintaining glucose balance, which regulates the balance of glucose levels in plasma and liver by controlling uptake and storage process of glucose [62–64]. The homeostatic processes are regulated by hormone, including insulin and glucagon as the main regulators of glucose metabolism. Peripheral tissue consuming glucose, glycolysis, and storage of glycogen was accelerated by insulin under the fed conditions, while glucagon offsets the effect of insulin and increases hepatic glucose production under fasting conditions [65]. Recent works have showed that p300/CBP is a regulator of glucose homeostasis which is critical factor in metabolic diseases.

### 4.1. Fasting and Hepatic Gluconeogenesis

In the states of limited energy, such as fasting or prolonged calorie restriction, the role of liver is to provide the organization with glucose to maintain normal glucose levels, originally through breaking down glycogen (glycogenolysis) and then converting to gluconeogenesis [66]. Several transcription factors and coactivators are involved in the gluconeogenesis controlled by nutrition and hormone. During fasting, hepatic glucose production is crucial as a source of fuel that maintains the basic functions of other tissues, including skeletal muscle, red blood cells, and the brain [67, 68]. Glucagon plays vital role during fasting state, in part by activating gluconeogenesis through cAMP response element-binding protein (CREB) and CREB-regulated transcription coactivator 2 (CRTC2) [69–72]. The factors forkhead box protein O1 (FOXO1) can also promote the transcription of gluconeogenic enzyme genes such as glucose-6-phosphatase (G6Pase) and phosphoenolpyruvate carboxykinase (PEPCK1), which in turn leads to elevating gluconeogenesis [73].

**Figure 2:**
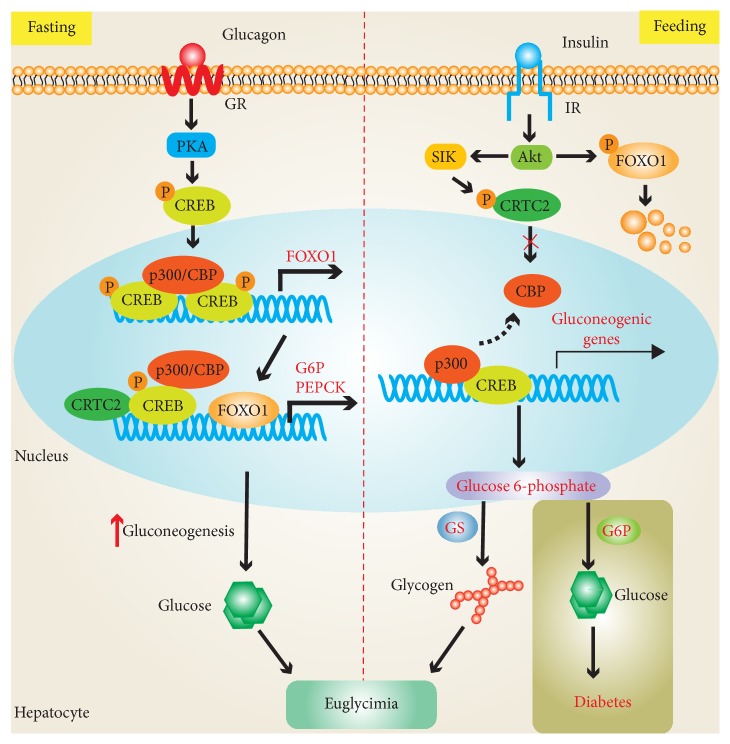
*Potential contributions of p300/CBP to the development of hepatic glucose homeostasis. In the fasting condition*, phosphorylation of CREB recruiting p300/CBP increases FOXO1 gene expression and, accordingly, FOXO1 induces PEPCK1 and G6Pase gene expression.* In the feeding condition*, p300 constitutively binds to the CREB coactivator complex maintaining glyconeogenesis and sustaining euglycemia through the activation of basal gluconeogenesis (CREB: cAMP response element-binding protein; FOXO1: hepatocyte nuclear factors forkhead box protein O1; G6Pase: glucose-6-phosphatase; PEPCK1: phosphoenolpyruvate carboxykinase).

FOXO1 is a significant downstream mediator of the insulin signaling pathway [22]. In the fed conditions, FOXO1 was phosphorylated by insulin through AKT, leading to its nuclear exclusion and degradation. FOXO1 levels were decreased in nuclear which led to suppressing the hepatic glucose production [74, 75]. On the other hand, FOXO1 was regulated via acetylation of p300 and CBP [76, 77]. Recently, Anne and colleagues showed that mRNA and protein levels of FOXO1 were elevated prominently in the mice livers after fasting. In the fasting conditions, phosphorylation of CREB at Ser-133 was activated by glucagon through cAMP-PKA pathway and led to recruitment of the p300/CBP and CRTC2 to CRE-containing genes including PEPCK1 and G6Pase. The elevated expression of gluconeogenesis was the major pathway in increasing hepatic gluconeogenesis [68, 78] and FOXO1 induced accordingly PEPCK1 and G6Pase gene expression [79, 80]. According to the information, the authors concluded that glucagon may elevate FOXO1 gene expression in the fasting state via p300/CBP. In this way, FOXO1 would further increase gluconeogenesis. Remarkably, silence of coactivator p300/CBP led to the decrease of mRNA and protein levels of FOXO1. In addition, suppression of histone acetyltransferase activity of p300/CBP prominently reduced mRNA and protein levels of FOXO1 in liver of fasting mice and fasting blood glucose levels. By characterization of* Foxo1* gene promoter, p300/CBP upregulated the expression of* Foxo1* gene via binding to tandem CREs in the proximal promoter region of* Foxo1* gene [22]. Taken together, these results indicate that p300/CBP may play a critical role in regulating gluconeogenesis in the fasting state (Figure 2).

### 4.2. Nutrient Supply and Hepatic Glucose Homeostasis

Hepatic glycogen breakdown maintains normal blood glucose level during early fasting; proper hepatic glycogen synthesis in the feeding/postprandial states is crucial. It has been known that gluconeogenesis is critical for hepatic glycogen synthesis. It was reported that depletion of hepatic p300 decreased glycogen synthesis and storage, which led to relative hypoglycemia [81]. Previous investigations have known that insulin suppressed gluconeogenesis by phosphorylating CBP at S436, which led to disassemble the CREB-CBP complex. However, p300 lacks the corresponding S436 phosphorylation site at CBP. In a phosphorylation-competent p300G422S knock-in mouse model, the authors showed that mutant mice decreased markedly hepatic glycogen content in the tracer incorporation assay under the postprandial state [81]. Therefore, p300 has a critical role in glycogen synthesis by maintaining basal gluconeogenesis.

Reports from decades ago have suggested that the gluconeogenic pathway accounted for 50%–70% of newly synthesized glycogen [82]. In the perfused rat liver or in primary rat hepatocytes, physiologic concentration of glucose had minimal effects on the glycogen synthesis when glucose was the sole substrate; however, efficient glycogen synthesis occurred when gluconeogenic precursors were added [83, 84]. Studies from rat, mouse, and dog using radiotracer-labeling techniques have firmly established that the gluconeogenic pathway contributed substantially to hepatic glycogen formation during postprandial state [85–87]. Human studies reached the same conclusion [88]. These studies indicate that a significant amount of gluconeogenesis still occurs even in the presence of elevated blood glucose levels and that physiologic hyperinsulinemia does not completely inhibit net gluconeogenic flux [85–89]. Therefore, in the postprandial and fed states hepatic gluconeogenesis is crucial for converting gluconeogenic precursors into glucose that can be stored as glycogen or released into blood, particularly when fed a high protein diet [90].

CBP and p300 are closely related proteins, but p300 lacks S436 phosphorylation site found on CBP that cannot be phosphorylated at this site. Recently, the authors found p300 constitutively bound to hepatic CREs such as found in the* Ppargc1* gene promoter and maintains basal gluconeogenesis in the fed and postprandial states [91]. In the fed state, HGP was partly inhibited by insulin via the phosphorylation of CBP at Ser-436. Due to the phosphorylation event, CBP and CRTC2 disassembled from the CREB complex [78]. In addition, the phosphorylation of p300 on Ser-79 and CBP on Ser-78 reduced their histone acetyltransferase activity and acetylation of CRTC2 via insulin and promoted nuclear exclusion and degradation of CRTC2 in the cytoplasm [79]. Indeed, p300 constitutively bound to the CRE site such as the* Ppargc1* gene promoter in both the fasting and feeding states. Thus, the activation of gluconeogenic pathway by constitutive binding of p300 to the CREB coactivator complex maintains glyconeogenesis and sustains euglycemia through the activation of basal gluconeogenesis in the postprandial state [81] (Figure 2).

Given that diabetic patients have increased hepatic gluconeogenesis [92, 93], one might predict that hepatic glycogen storage would be increased. In fact, however, previous studies have shown that diabetic patients have lower hepatic glycogen content due to decreased glycogen synthase (GS) and/or increased glycogen phosphorylase activity [92, 93]. This discrepancy might be explained by an increase in glucose 6-phosphatase in diabetic patients [94, 95], which would increase the conversion of glucose 6-phosphate to glucose rather than being used for glycogen synthesis. Furthermore, p300 maintained constitutive activation of gluconeogenesis even in the postprandial state, which is due to the fact that p300 activity is not regulated by insulin [91]. The latter finding may explain rodent and human data, which suggests that gluconeogenesis is unable to be completely inhibited even when high serum insulin levels are achieved in insulin-sensitive normal animals and human subjects.

## 5. Hepatic Inflammatory Response and Fibrosis

### 5.1. NF-*κ*B in Proinflammatory Response

**Figure 3:**
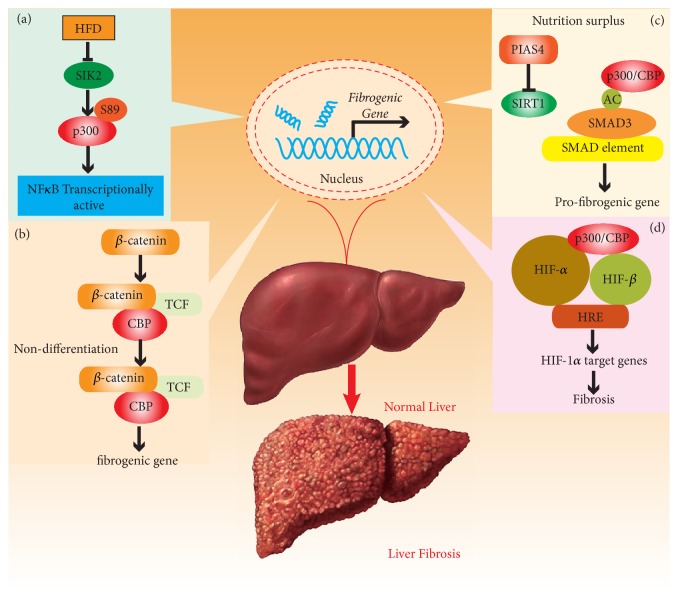
*Potential contributions of p300/CBP to the development of proinflammatory response and liver fibrosis*. (a) p300 HAT activity inhibited by SIK2 activates phosphorylated NF-*κ*B in HFD. (b) CBP/*β*-catenin-mediated transcription in fibrogenic genes expression. (c) p300/CBP acetylates K378 in the MH2 domain of SMAD3 and enhances the transcriptional activity of SMAD3. (d) p300/CBP interacts with HIF-1*α* forming the HIF-1*α*-p300/CBP complex and then binding to the hypoxia response element (HRE) to activate the transcription of genes involved in fibrogenesis. (SIK2: salt-inducible kinase 2; NF-*κ*B: nuclear factor kappa B; HIF-1*α*: hypoxia-inducible factor-1alpha).

It is well known that immunity and metabolism are closely related [96]. It has been reported that chronic low-grade systemic inflammation plays a critical role in metabolic disorders such as obesity, insulin resistance, and T2DM [97]. In this condition, activation of nuclear factor kappa B (NF-*κ*B) is crucial effector that induces inflammatory responses. A study showed that p300/CBP had a significant role in inflammation by relating with the phosphorylated NF-*κ*B [98]. Since then, a recent study found that salt-inducible kinase 2 (SIK2) activity, activated by insulin and inhibited by glucagon, was decreased in mice livers fed on a high-fat diet (HFD) [99]. SIK2 can suppress p300 function through the inhibition of its HAT activity via direct phosphorylation at Ser89 [19]. SIK2 reduced expression led to an inflammation characterized by increased secretion of IL-6 and TNF-*α* and stimulation of NF-*κ*B activity via downregulation of p300 HAT activity. HFD exposure activated the hepatic NF-*κ*B signaling pathway, which caused insulin resistance, thereby linking inflammation to insulin resistance [24]. Therefore, this finding indicated that SIK2 activators or p300 inhibitors, by decreasing acetylation of p300, may offer novel therapeutic measures to treat hepatic diseases such as liver fibrosis (Figure 3(a)).

### 5.2. CBP/*β*-Catenin in Liver Fibrosis

Liver fibrosis is a common pathology of various progressive chronic liver diseases [100]. Under nutrition surplus conditions, some different types of cells, such as hepatic stellate cells (HSCs) and portal fibroblast cells, trans-differentiate into profibrogenic myofibroblasts, which promote the deposition and production of extracellular matrix (ECM) proteins [101]. Although it is thought to be a host defense mechanism, excessive fibrosis formation may destroy the normal liver structure and function, leading to the end-stage liver diseases such as cirrhosis and hepatocellular carcinoma [102].

Wnt/*β*-catenin is involved in almost every aspect of embryonic development as well as the pathogenesis of many human diseases and is also involved in self-renewal in adult tissues such as liver and lung repair after an injury [103–105]. Recently, Wnt/*β*-catenin has been reported to be associated with organ fibrosis, suggesting that they may be new therapeutic targets in liver fibrosis [106, 107]. HSCs represent a primary fibrogenic cell type in the liver [108]. HSCs is activated in a liver fibrosis, which change their phenotype from quiescent retinoid storing HSCs to collagen producing and contractile myofibroblast-like cells [109]. Following activation by upstream signaling from Wnt/*β*-catenin translocates to the nucleus. Nuclear *β*-catenin recruits the Kat3 transcriptional coactivators, CBP or p300, to stimulate the transcription of its target genes, and distinct roles have been reported for CBP and p300 [110, 111]. CBP/*β*-catenin-mediated transcription is crucial for proliferation/nondifferentiation, whereas p300/*β*-catenin-mediated transcription activates differentiation [112]. A recent study found that CBP/*β*-catenin was the major interactor and regulator of liver fibrosis [25]. The effects of PRI-724, an inhibitor of CBP/*β*-catenin, on liver fibrosis, were examined through using a mouse liver fibrosis models induced by carbon tetrachloride (CCl4) or bile duct ligation (BDL). PRI-724 treatment decreased the liver fibrosis induced by CCl4 or BDL. Mechanically, the authors investigated the contribution of CBP/*β*-catenin to the liver fibrosis of mice [25]. The results indicated that the inhibition of CBP/*β*-catenin suppressed liver fibrosis through the inhibition of HSC activation, which provided novel therapeutic possibilities for treating the liver fibrosis (Figure 3(b)).

### 5.3. STAT4(PIAS4)/SMAD3 Signaling Pathway and Liver Fibrosis

In the cells produced by ECM, fibrogenesis is determined by a network of growth factors, cytokines, and transcription factors [113]. Transforming growth factor (TGF-*β*) is by far the most extensively studied in the liver to promote fibrogenesis factor, mainly through the SMAD family of transcription factors [114]. SMAD3 is an effective profibrogenic transcription factor, which has long been reported as a regulator of the signaling pathway downstream of transforming growth factor [115–117]. SMAD3 activity can be regulated by its posttranslational modifications. For example, serine phosphorylation is critical for SMAD3 dimerization and nuclear translocation [114].

Recent studies may demonstrate that p300/CBP functions as the regulator of liver fibrosis through modulating SMAD3 activity. Researchers found that* PIAS4* silencing using short hairpin RNA (shRNA) reduced high-fat high-carbohydrate (HFHC) diet induced liver fibrosis in mice [118]. Mechanistically, the recruitment of SMAD3 to the promoter regions of profibrogenic genes was suppressed by silencing PIAS4, which led to dampening SMAD3 acetylation likely by upregulating expression of SIRT1 [118]. In another study, PIAS4 was activated by treating with high glucose in cultured primary mouse HSCs while at the same time suppressing SIRT1. SIRT1 promoter activity was repressed by the overexpression of PIAS4 which resulted in SMAD3 hyperacetylation and increased SMAD3 binding to fibrogenic gene promoters [26]. Coincidentally, Inoue et al. found that p300/CBP acetylated K378 in the MH2 domain of SMAD3 and enhanced the transcriptional activity of SMAD3 [119]. Moreover, a recent study showed that high glucose increases the activity of the transcriptional coregulator p300, which increased TGF-*β* activity via SMAD3 acetylation [120]. Taken together, these findings suggest the possibility that p300/CBP may contribute to liver fibrosis by promoting acetylation of SMAD3 in high glucose condition. However, further studies are necessary to verify this hypothesis (Figure 3(c)).

### 5.4. HIF1-*α* Recruits P300/CBP in Liver Fibrosis

Hypoxia-inducible factor-1alpha (HIF-1*α*) is a crucial transcription factor in response to hypoxic stress by modulating expression of genes involved in maintaining oxygen homeostasis [121]. Recently, the upregulation of HIF-1*α* was documented to be concerned with activation of HSCs and liver fibrosis [122]. The function of HIF-1*α* in the development of liver fibrosis has certified by the experiments used in HIF-1*α* liver-conditional knockout mice, which suggested that HIF-1*α*-recruitment preceded fibrosis and that the silencing of HIF-1*α* led to a remarkable decrease of ECM deposition in hepatocyte [122]. Recently, the activation of HIF-1*α* by stimulation of hypoxia was demonstrated to be a promoter of HSC/myofibroblast-like cells (MFs) migration in the human HSCs. In addition, the silencing of HIF-1*α* resulted in primary inhibition of HSC/MFs migration, which suggested a significant function of HIF-1*α* in the development of liver fibrosis [123].

A recent study showed that inhibiting HIF activity through the disassembly of the HIF-1*α*-p300/CBP protein-protein interaction was considered to be a potential therapeutic target for hepatic fibrosis [27]. HIF-1*α* is degraded at the posttranscriptional level via interaction with the von Hippel-Lindau protein, in normoxic conditions [124]. Moreover, HIF-1*α* is also hydroxylated at Asn803 by factor inhibiting HIF1, blocking the interaction of HIF-1*α* with the p300/CBP coactivator protein [125]. Nevertheless, under hypoxic conditions, HIF-1*α* accumulates and dimerizes with HIF-1*β*, forming a protein heterodimer that complexes with p300/CBP in the nucleus. The HIF-1*α*-p300/CBP complex then binds to the hypoxia response element (HRE) to activate the transcription of genes involved in fibrogenesis [126]. Thus, a measure to repress hypoxic responses in fibrosis may be to target the protein-protein interaction between HIF-1*α* and p300/CBP. Wu et al. reported, for the first time, that the aminocoumarin antibiotic, novobiocin, directly blocked the protein-protein interaction between HIF-1*α* and p300/CBP. Novobiocin directly bound to HIF-1*α* C-terminal activation domain (CTAD), suppressed he cruitment of transcriptional coactivator p300/CBP, leading to decrease of hypoxically regulated genes [127]. Ultimately, this repressed liver fibrosis. These results indicated a novel mechanism of inhibiting HIF-1*α*-p300/CBP complex which has the potential for innovative therapeutic use in liver fibrosis therapy (Figure 3(d)).

## 6. Nutritional Therapeutic Approaches

Curcumin is a kind of polyphenol that was found in the popular spice turmeric. Curcumin's biological functions has been widely studied. It is well known that it has features like antioxidant, anti-inflammatory, and anticancer properties [128]. Curcumin was found to be a HAT inhibitor even as late as 2004. There are already more than 5000 publications involving certain aspects of curcumin to date. More importantly, curcumin is a certain inhibitor of p300/CBP [129]. NF-*κ*B activity and the production of interleukin-6 and TNF-*α* were promoted by the high glucose-induced elevations in p300 activity. And the high glucose-induced elevations in p300 activity were abrogated by curcumin [130]. Curcumin derivative attenuates liver fibrosis [131]. The mechanism of curcumin conveying this protection is not well understood, but its effects via inhibiting the activity of p300 has the possibility of playing a critical role in the process of protection. A polyphenolic phytoestrogen resveratrol has shown the same features as curcumin [132]. Moreover, resveratrol is a natural polyphenol that can be found in grapes and red wine known to activate the deacetylase activity of silent information regulator 1 (SIRT1) and that would deacetylate and inhibit the FOXO1 [133]. But, the histone acetyltransferase p300 has been known to be able to acetylate and increase nuclear localization and transactivating activity of FOXO1 [134]. For that reason, resveratrol may orchestrate glucose homeostasis and diabetes by increasing deacetylation of FOXO1 through SIRT1 to suppress the acetylation of p300. Another inhibitor of p300 is procyanidins. Procyanidins from grape seed extracts possess an efficient anti-HAT activity. In a current study, the authors found that Pro-B3 (procyanidins B3) exhibits the strongest inhibition against p300 HAT of all catechin derivatives [135]. A recent study demonstrated that procyanidin was related to significant protection against serious liver injury in mice which was caused by the induction of CCl_4_ which was the most widely used chemical liver poisoning that causes experimental liver fibrosis [136]. As mentioned above, p300/CBP plays a significant role in regulating liver fibrosis. Thus, it can be foretold that curcumin, resveratrol, and procyanidins could serve as protection of liver diseases by inhibiting p300 HAT.

With advance in our developing knowledge of the contribution of epigenetic modifications to the development of metabolic syndrome, the roles of dietary constituents with HAT and HDAC regulating properties, such as curcumin, resveratrol, and procyanidins, in the pathogenesis of the metabolic diseases have been identified. Nevertheless, the molecular mechanisms of potential inhibitory roles still need further researches, which would reveal the mechanism of effect of these dietary constituents in the prevention of liver diseases.

## 7. Conclusions and Future Perspectives

It is obvious that the field of nutritional epigenetics is further clarifying the mechanisms of gene-nutrient interaction. It provides the role of nutrition in defining phenotype from genotype [137]. As a result, there is great meaning in identifying epigenetic-based therapeutic strategies as a method to prevent the development of liver diseases. In animal and cellular researches, p300/CBP, via acetylation of several substrates, allows a variety of interactions with constituents of significant metabolic pathways like lipogenesis, lipid export, gluconeogenesis, and inflammatory pathways. Further studies suggested that p300/CBP possessed histone acetyltransferase activity and were involved in many cellular processes.

Glucose and lipid are the primary energy sources. They are also pivotal components of organic metabolism in mammals. Inappropriate diet can influence the metabolic rate directly. And it can alter the body's homeostasis. The underlying changes in energy storage and utilization would be shown as metabolic syndrome including obesity and high blood pressure and high blood glucose, which are predisposing features that significantly increase the risk for liver diseases and type 2 diabetes [138]. Plenty of reports have suggested that supplementation of some components such as curcumin, resveratrol, and procyanidins, can help moderate high-fat-induced inflammation, obesity, glucose intolerance, and insulin insensitivity. Although the molecular mechanisms underlying the inhibitory roles of them in the development of high-fat-induced metabolic syndrome are unclear, the effects are, at least in part, through modifications in the expression and activity of P300/CBP. In this review, it is further clear that p300/CBP plays an essential role in the progress of hepatic energy homeostasis. For instance, under the high glucose conditions, p300/CBP upregulates the expression of lipogenic genes (FAS, ACC, ACLY, and SCD1) by acetylating SREBP1C and ChREBP, which promotes its stability and recruitment to its lipogenic target gene promoters. On the other hand, p300/CBP is involved in hepatic gluconeogenesis produces no matter in the fasting or feeding condition; however, their purposes are all to keep the stability of glucose homeostasis. These evidences demonstrate that p300/CBP plays different roles in maintaining energy homeostasis. In metabolic disease states, p300 actives gluconeogenic pathway by constitutive binding of the CREB coactivator complex in the continuous supply of nutrients. This may provide an explanation for why obese patients are prone to have diabetes.

What is more, as super enhancer inducers, under the condition of overnutrition, p300/CBP stimulates expression of key genes in liver fibrosis. p300/CBP acts on liver cellular targets (SMAD3, *β*-catenin, HIF-*α*, and NF*κ*B) to stimulate fibrogenic genes expression. Thus, inhibition of p300/CBP activity is a versatile strategy to treat such liver fibrosis and has the potential to be impactful for a variety of patients with heterogeneous drivers of liver fibrosis. For instance, curcumin has long been identified as a p300/CBP inhibitor of histone acetyltransferase activity, thus alleviating fibrosis may be a therapeutic strategy. However, it is currently unclear whether curcumin, resveratrol, or procyanidins mediates their effect by p300/CBP altering the acetylation state of metabolic enzymes. More work is needed to better understand the mechanisms by which regulation of p300/CBP affects downstream protein interactions and signaling pathways. Nonetheless, targeting the activity of the significant transcriptional coactivators p300/CBP may represent a potential avenue for the treatment of liver fibrosis in later researches (Table 1).

Even transcriptional coactivator p300/CBP for hepatic energy homeostasis and fibrosis has been identified and studied, to explain the molecular mechanism of the transcriptional regulation of lipogenesis, gluconeogenesis, and fibrogenesis, more issues yet to be fully elucidated. Initially, how do multiple transcription factors activate genes in a cooperating way? Secondly, what enzymes regulated by p300/CBP in response to nutrients supply or deprive are involved in posttranslational events? Thirdly, except curcumin, resveratrol, and procyanidins, are there any other inhibitors of p300/CBP that are related in the alleviation of liver fibrosis? Answering these questions may be fatal for assessing the potential specificity and efficacy of p300/CBP modulation into the therapeutic strategy of liver diseases.

## Figures and Tables

**Table 1:** Overview of p300/CBP regulates liver lipid, glucose metabolism, and liver fibrosis through regulating different targets.

Progress	Activity	Targets	Effect	Nutritional state	Metabolic response	Reference
Lipid metabolism	Acetylation	FXR	Inhibitory	High glucose and insulin	Lipogenesis↑ Gluconeogenesis↑	[17, 18]
	Acetylation	ChREBP	Stimulatory	High glucose and insulin	Lipogenesis↑	[19]
	Acetylation	SREBP1c	Stimulatory	High glucose and insulin	Lipogenesis↑	[20]
	Binding	DDX3	Stimulatory	/	Lipid export↑	[21]

Glucose metabolism	Acetylation	FOXO1	Stimulatory	Fasting state	Gluconeogenesis↑	[22]
	Binding	CREB	Stimulatory	Feeding state	Gluconeogenesis↑	[23]

Liver fibrosis	Binding	NF-*κ*B	Stimulatory	High-fat diet	Proinflammatory response↑	[24]
	Binding	*β*-Catenin	Stimulatory	/	Liver fibrosis↑	[25]
	Acetylation	SMAD3	Stimulatory	High-fat high-carbohydrate	Liver fibrosis↑	[26]
	Binding	HIF-1*α*	Stimulatory	/	Liver fibrosis↑	[27]

## Abbreviations

T2DM: Type 2 diabetes mellitus
NAFLD: Nonalcoholic fatty liver disease
PTMs: Posttranslational modifications
HATs: Histone acetyltransferases
HDACs: Histone deacetylases
SREBP1C: Sterol regulatory element-binding transcription factor 1
FAS: Fatty acid synthase
ACC: Acetyl-CoA carboxylase
ChREBP: Carbohydrate-responsive element-binding protein
ACLY: ATP citrate lyase
SCD1: Stearoyl-CoA desaturase
PKA: Protein kinase A
FXR: Bile acid receptor
MTP: Microsomal triglyceride transfer protein
HNF4: Hepatocyte nuclear factor 4
CREB: cAMP response element-binding protein
CRTC2: CREB-regulated transcription coactivator 2
FOXO1: Hepatocyte nuclear factors forkhead box protein O1
G6Pase: Glucose-6-phosphatase
PEPCK1: Phosphoenolpyruvate carboxykinase
GS: Glycogen synthase
NF-*κ*B: Nuclear factor kappa B
SIK2: Salt-inducible kinase 2
HSCs: Hepatic stellate cells
ECM: Extracellular matrix
BDL: Bile duct ligation
TGF-*β*: Transforming growth factor
shRNA: Short hairpin RNA
HIF-1*α*: Hypoxia-inducible factor-1alpha
MFs: Myofibroblast-like cells
HRE: Hypoxia response element
CTAD: C-terminal activation domain
SIRT1: Silent information regulator 1.
